# Superionic Liquids in Conducting Nanoslits: Insights from Theory and Simulations

**DOI:** 10.1021/acs.jpcc.0c10836

**Published:** 2021-03-01

**Authors:** Yaroslav Groda, Maxym Dudka, Alexei A. Kornyshev, Gleb Oshanin, Svyatoslav Kondrat

**Affiliations:** † Department of Mechanics and Engineering, Belarusian State Technological University, Sverdlova str., 13a, 220006 Minsk, Belarus; ‡ Institute for Condensed Matter Physics of the National Academy of Sciences of Ukraine, 1 Svientsitskii st., 79011 Lviv, Ukraine; § L^4^ Collaboration & Doctoral College for the Statistical Physics of Complex Systems, Leipzig-Lorraine-Lviv-Coventry, D-04009 Leipzig, Europe; ∥ Institute of Theoretical Physics, Faculty of Physics, University of Warsaw, Pasteura 5, 02-093 Warsaw, Poland; ⊥ Department of Chemistry, Molecular Sciences Research Hub, White City Campus, W12 0BZ London, United Kingdom; # Thomas Young Centre for Theory and Simulation of Materials, Imperial College London, South Kensington Campus, SW7 2AZ London, United Kingdom; ∇ Sorbonne Université, CNRS, Laboratoire de Physique Théorique de la Matière Condensée, LPTMC (UMR CNRS 7600), 75252 Paris Cedex 05, France; ○ Institute of Physical Chemistry, Polish Academy of Sciences, Kasprzaka 44/52, 01-224 Warsaw, Poland; ◆ Max-Planck-Institut für Intelligente Systeme, Heisenbergstraße 3, D-70569 Stuttgart, Germany; ¶ IV. Institut für Theoretische Physik, Universität Stuttgart, Pfaffenwaldring 57, D-70569 Stuttgart, Germany

## Abstract

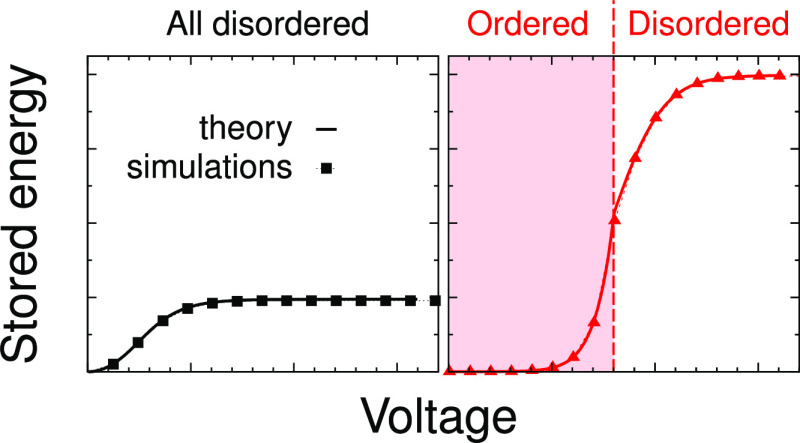

Mapping the theory of charging supercapacitors with nanostructured electrodes on known lattice models of statistical physics is an interesting task, aimed at revealing generic features of capacitive energy storage in such systems. The main advantage of this approach is the possibility to obtain analytical solutions that allow new physical insights to be more easily developed. But how general the predictions of such theories could be? How sensitive are they to the choice of the lattice? Herein, we address these questions in relation to our previous description of such systems using the Bethe-lattice approach and Monte Carlo simulations. Remarkably, we find a surprisingly good agreement between the analytical theory and simulations. In addition, we reveal a striking correlation between the ability to store energy and ion ordering inside a pore, suggesting that such ordering can be beneficial for energy storage.

## Introduction

Confined ionic liquids show an exciting physics and play an increasingly important role in technology, finding their applications in various electrochemical devices, such as capacitive energy storage^1−4^ and deionization systems,^5−7^ heat-to-energy converters,^8−11^ etc. Electrochemical capacitors, or supercapacitors, for instance, store energy through charge separation between their cathode and anode.^12^ Since the amount of the stored charge scales with the contact area between the electrode and an electrolyte, a method to boost the energy storage is to use electrodes with large volume-filling surface area, which are provided by highly porous electrodes, particularly those containing subnanometer pores. Such pores, which can admit just a single layer or row of ions, deliver the highest achievable capacitance,^13−15^ allegedly due to a superionic state emerging in a narrow conducting confinement.^16^ In the superionic state, the electrostatic interactions between the ions are strongly screened,^16−19^ allowing a tighter packing of counterions and easier unbinding of “ion pairs”, thus enhancing the capacitance.^16,20^

The simplest model for charge storage in subnanometer pores is a classical antiferromagnetic spin-1 Ising model with nearest-neighbor interactions in external field.^21^ In this model, cations and anions correspond to ±1 spins and the external magnetic field corresponds to the potential drop between the electrode and bulk electrolyte. In one dimension (1D), the Ising model mimics a single-file pore and obeys the well-known exact solution,^22^ allowing physically appealing analyses of the charging behavior.^21^ In deviance of its simplicity, this model turns out to capture the essential physics of charging nanoscale pores quite well.^23,24^ More complex 1D models have also been used in various contexts.^25−31^

Mapping the confined ions onto 1D lattice spin models has an advantage that such models are often exactly solvable in external field, which corresponds to the cell voltage in electrochemical devices. Such quasi-1D pores are typical for electrodes based on open carbon nanotubes (CNTs) or on a forest of closed CNTs. For the state-of-the-art graphene-based^32−37^ and MXene^38−40^ electrodes, slit pores are a better approximation to the reality. New physics emerges in such pores and particularly exciting is the possibility to observe phase transitions, which cannot occur in 1D systems.^41,42^

Two-dimensional lattice models are widely used in the literature to study confined or adsorbed ions, but they are mainly handled by simulations.^43−46^ Recently, a three-component model has been proposed^47,48^ for ionic liquids in narrow conducting slits, which can be mapped onto the Blume–Capel (BC) model, well known in the theory of magnetism.^49−53^ Similarly to the Ising model, the BC model has been solved exactly only in 1D. In refs (47, 48), this model was treated with a Bethe-lattice approximation, which allowed analytical insights into the dependence of charging on applied voltage and other parameters. A natural question is, however, how accurate and reliable these analytical results are. In a broader context, the Bethe-lattice approximation is a convenient analytical tool that is frequently used to tackle various models of statistical physics, and hence the above question is of a general relevance.

Herein, we assess the accuracy of the Bethe-lattice approximation with Monte Carlo (MC) simulations of the model of ref (47) on the square, honeycomb and, in a few cases, triangular lattices. In addition, with the Bethe-lattice calculations, we discuss which of these three lattices is more likely to be realized in off-lattice simulations and experiments. With MC simulations, we gain additional insights into the structural transformations of ionic liquids in narrow slit confinements and investigate how ordering of ions is related to energy storage.

Of course, our model does not account for many properties of real electrodes and electrolytes, such as the carbonic nature of pore walls, dispersion interactions, ion and solvent polarizability, etc. (see Section II.C in ref (48) for a detailed discussion of model limitations). In this sense, the model is probably too idealized to directly compare with experiments using the currently available microporous carbons, although it might be possible to do so with future nanostructured electrodes. Nevertheless, we expect some qualitative features of the charging behavior and stored energy–ion ordering relations, revealed in this article, to be generic and model-independent.

## Model and Methods

### Model

**Figure 1:**
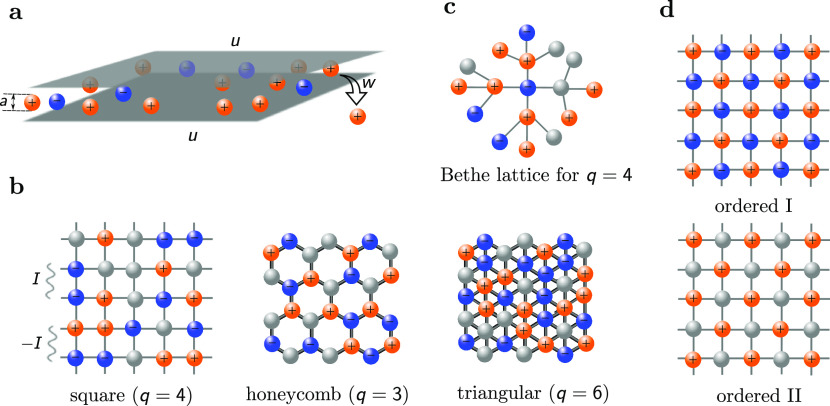
Model for ions in a narrow slit nanopore. (a) Ions are confined into a narrow metallic slit. Ion diameter *a* = *a*_±_ is the same for cations and anions. Potential difference *u* is applied at the pore walls with respect to the bulk electrolyte (not shown). The energy of transfer of ions from the pore into the bulk *w* = *w*_±_ is the same for both ions (see text). (b) Arrangement of ions on square, honeycomb, and triangular lattices, used in Monte Carlo (MC) simulations of model (1). Blue and orange spheres represent ions, and gray spheres represent void/solvent. Interaction energy between the same type of ions is *I*, and that between oppositely charged ions is −*I* (see eq 1). (c) Fragment of the Cayley tree with coordination number *q* = 4, corresponding to the square lattice. (d) Ordered phases on the square lattice. Ordered phase I consists of an equal amount of cations and anions occupying two sublattices. In ordered phase II, the counterions occupy one sublattice while the other sublattice is occupied by solvent/void.

To study charge storage in ultranarrow slit nanopores that are so narrow that they admit only one layer of ions, we consider a model Hamiltonian defined on a two-dimensional lattice^47,48^ (Figure 1)
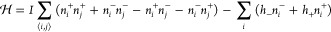
1Here, *n*_*i*_^+^ = 1 (*n*_*i*_^–^ = 1) means that site *i* is occupied by a cation (an anion) and *n*_*i*_^+^ = *n*_*i*_^–^ = 0 means that site *i* is vacant or occupied by solvent (configuration *n*_*i*_^+^ = *n*_*i*_^–^ = 1 is prohibited due to hard-core exclusion); ⟨*ij*⟩ denotes nearest-neighbor sites and *I* > 0 is the interaction energy between two neighboring ions. For ions confined between two metallic plates *I* ≈ ϕ (*a*), where *a* is the lattice constant (≈the ion diameter) and^16^
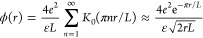
2with *K*_0_(*x*) being the modified Bessel function of the second kind of order zero, *L* the slit width, *e* the elementary charge, ε a dielectric constant inside a pore, which comes from electronic and rotational degrees of freedom of ions and due to a solvent, if present. The value of ε is not precisely known, but we expect it to vary from 2 for simple ions to 5 or more for more complex, bulky ions or in the presence of a solvent.

Equation 2 gives coupling constants *I* that vary from just a *k*_B_*T* for large ions and narrow slits (*L*/*a* ≈ 1) to a few *k*_B_*T* for small ions or wide slits. *I* increases for increasing *L* at constant *a* and decreases for increasing *a* at constant *L* (Fig. S1). Note that we consider *1 < L*/*a* < 2, that is, the confined ions form a single layer. For *L*/*a* values close to but smaller than 2, the next-nearest interactions may become important.^48^ It will be interesting to study such effects in future work.

In eq 1, “external” fields *h*_±_ are
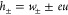
3where *u* is the applied potential (with respect to the bulk electrolyte outside of the pore) and *w*_±_ is the energy of transfer of a ± ion from an empty into the bulk, which includes the image forces and other interactions of the ions with the pore walls.^16^ We note that the definition used here differs by sign from the re-solvation energy used in other works.^16,54,55^ We assume *w*_+_ = *w*_–_ = *w* and note that the results for an asymmetric ionic liquid (*w*_+_ ≠ *w*_–_) can be obtained by shifting the applied voltage by −(*w*_+_ – *w*_–_)/2*e* and taking the transfer energy equal to (*w*_+_ + *w*_–_)/2.

Based on quantum-mechanical calculations and molecular dynamics simulations,^56^ Lee et al.^57^ have estimated that for slit pores, the nonelectrostatic contribution *w*_non_ to *w* ranges from −5 to 45 *k*_B_*T*. The ion’s self-energy *w*_self_ (due to image charges^16^) is negative and varies from a few negative *k*_B_*T*’s for wide slits to about −50 *k*_B_*T* for narrow slits (Figure S2). Thus, the total ion’s transfer energy, *w* = *w*_self_ + *w*_non_, can span a wide range of values from negative to positive around zero. We note that changing the slit width or temperature affects both β*I* and β*w*.

### Monte Carlo Simulations

We performed two-dimensional (2D) Monte Carlo (MC) simulations of model (1) on square, honeycomb, and triangular lattices (Figure 1). Periodic boundary conditions were applied in both directions. The lattice size was 32 × 32 sites in all simulations, but in selected cases, we carried out simulations for smaller and larger lattices to study the finite-size effects. We observed significant deviations only in the vicinity of second-order transitions, as one might expect (Figures S4, S6, and S8). The system was equilibrated with 2 × 10^5^ MC steps, each consisting of *M* = 32^2^ single-spin updates, and the averaging was performed over 8 × 10^5^ MC steps.

### Bethe-Lattice Solution

The free energy of the system is
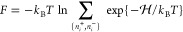
4where the sum runs over all possible configurations of the occupation numbers *n*_*i*_^+^, *n*_*i*_^–^ on a given lattice. Within the Bethe-lattice approach, eq 4 is evaluated on a Bethe lattice with the same coordination number *q* as of the original lattice (Figure 1c). The partition function on the Bethe lattice was computed exactly. For details of the calculations, see ref (47) (nonpolarized electrodes) and ref (48) (voltage dependence, *q* = 3). In this work, we took *q* = 4, 3, and 6, corresponding to square, honeycomb, and triangular lattices used in the MC simulations.

### Ordered and Disordered Phases

Recently, ref (48) has demonstrated a rich phase behavior of model (1) (with *q* = 3 neighbors), involving direct and re-entrant symmetry-breaking phase transitions between “ordered” and “disordered” phases. The disordered phase is a homogeneous mixture of ions and voids, while ordered phase means that the ions of one type predominantly occupy one of the two sublattices. We will see that at low applied voltages, the ordered phase consists of an equal amount of cations and anions (ordered phase I) so that the pore is neutral or only weakly charged. In the ordered phase at high potentials, one sublattice is occupied by counterions and the other sublattice is mostly free of ions (ordered phase II). Typical ion configurations in these phases are schematically shown in Figure 1d.

### Thermodynamic Characteristics and Charging

Quantities directly accessible from the Bethe-lattice calculations and MC simulations are the average number of ions ⟨*n*^±^⟩ in the pore. It is convenient to discuss charging in terms of the total ion density and the accumulated charge
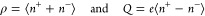
5Note that by definition, these quantities are measured per lattice site and to transform them to the corresponding quantities per surface area, one has to divide them by the area per site, which is *a*^2^, 3*a*^2^√3/4, and *a*^2^√3/2 for the square, honeycomb, and triangular lattices, respectively.

Having calculated *Q*(*u*), one can compute the differential capacitance
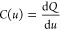
6aIn MC simulations, however, it is more convenient to evaluate the capacitance from charge fluctuations^58^
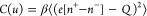
6bHaving the capacitance, one can calculate the energy stored in a nanopore at potential difference *u*
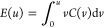
7Similar to charge and density, the capacitance and stored energy are measured per lattice site.

We shall also calculate the charging parameter^59,60^
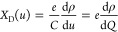
8awhich describes charging mechanisms taking place at applied voltage *u*. It is not difficult to see that *X*_D_ can be expressed in terms of charge-density fluctuations
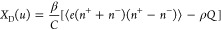
8bWe have used eq 8b to compute *X*_D_ in MC simulations.

Finally, we shall study the behavior of density fluctuations, related to the isothermal compressibility
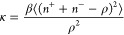
9This quantity describes how liable the system is to compression during charging, which may be relevant to electroactuators^61^ and studies of electrode deformations.^62^

## Results and Discussion

### Phase Diagram for Nonpolarized Electrodes

**Figure 2:**
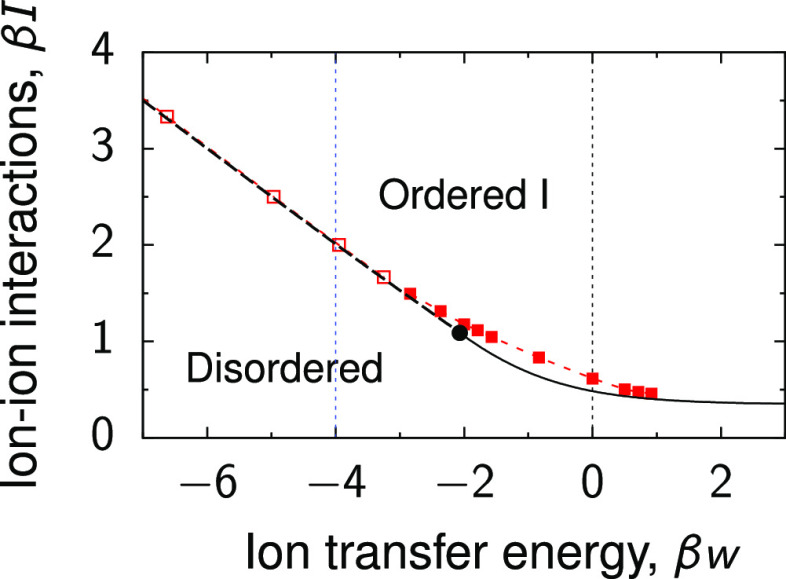
Phase diagram of a superionic liquid in a nonpolarized ultranarrow slit. The results of the Bethe-lattice calculations with coordination number *q* = 4 are shown by lines, and the square symbols denote the results of Monte Carlo simulations on a square lattice. The filled circle shows the tricritical point obtained within the Bethe-lattice approach. The black solid line and filled squares denote second-order continuous transitions, and the black dashed line and open squares show first-order discontinuous transitions between a disordered phase and the ordered phase I. For schematics of ordered and disordered phases, see Figure 1. The thin vertical lines show the values of the transfer energy chosen to study the charging behavior (Figures 3 and 4). For the phase diagram on the honeycomb lattice, see Figure S5.

Figure 2 shows a phase diagram spanned in the plane of ion–ion interaction energy *I* and ion transfer energy *w* for a nonpolarized electrode (i.e., potential difference *u* = 0 with respect to the bulk electrolyte). The phase diagram consists of disordered and ordered phases separated by the lines of first-order and second-order phase transitions, which meet at a tricritical point (filled circle in Figure 2). As discussed, the disordered phase is a homogeneous mixture of ions and solvent/voids, whereas the ordered phase (type I) consists of an equal amount of cations and anions occupying two sublattices of the square lattice (Figures 1d and S3). For the phase diagram on the honeycomb lattice, see Figure S5.

From MC simulations, the locations of the transitions were determined by analyzing the density profiles on two sublattices (Figure S3). While for the discontinuous transitions far from the tricritical point, this procedure leads to an excellent agreement with the Bethe-lattice calculations, for the second-order transitions, there are some deviations. In particular, in the MC simulations, the transitions are shifted toward higher values of β*I* (Figures 2, S3, and S4a). This is probably because the Bethe-lattice approach underestimates the effect of fluctuations in the ordered phase. Nevertheless, the agreement between the MC simulations and analytical Bethe-lattice calculations is remarkable.

The diagram in Figure 2 shows the states of systems characterized by different values of coupling constant *I* and transfer energy *w*. We now select a few typical systems from this diagram and investigate how they respond to a voltage applied to the pore with respect to the bulk electrolyte.

### Charging

Using the Bethe-lattice approach, ref (48) has demonstrated a complex charging behavior of model (1) for coordination number *q* = 3, corresponding to the honeycomb lattice. This behavior is essentially determined by the ion transfer energy *w* and can be divided into four zones related to the number of phase transitions that the system undergoes during charging. Here, with the Bethe-lattice approach, we find a qualitatively similar behavior for *q* = 4. We assess these Bethe-lattice results with MC simulations on the square lattice, which allow us to gain additional physical insights into the transformation of ionic structure under an applied voltage. These results are discussed below. The corresponding comparison for the honeycomb lattice is presented in the Supporting Information (Figures S7 and S9).

#### Positive Transfer Energy of Ions

**Figure 3:**
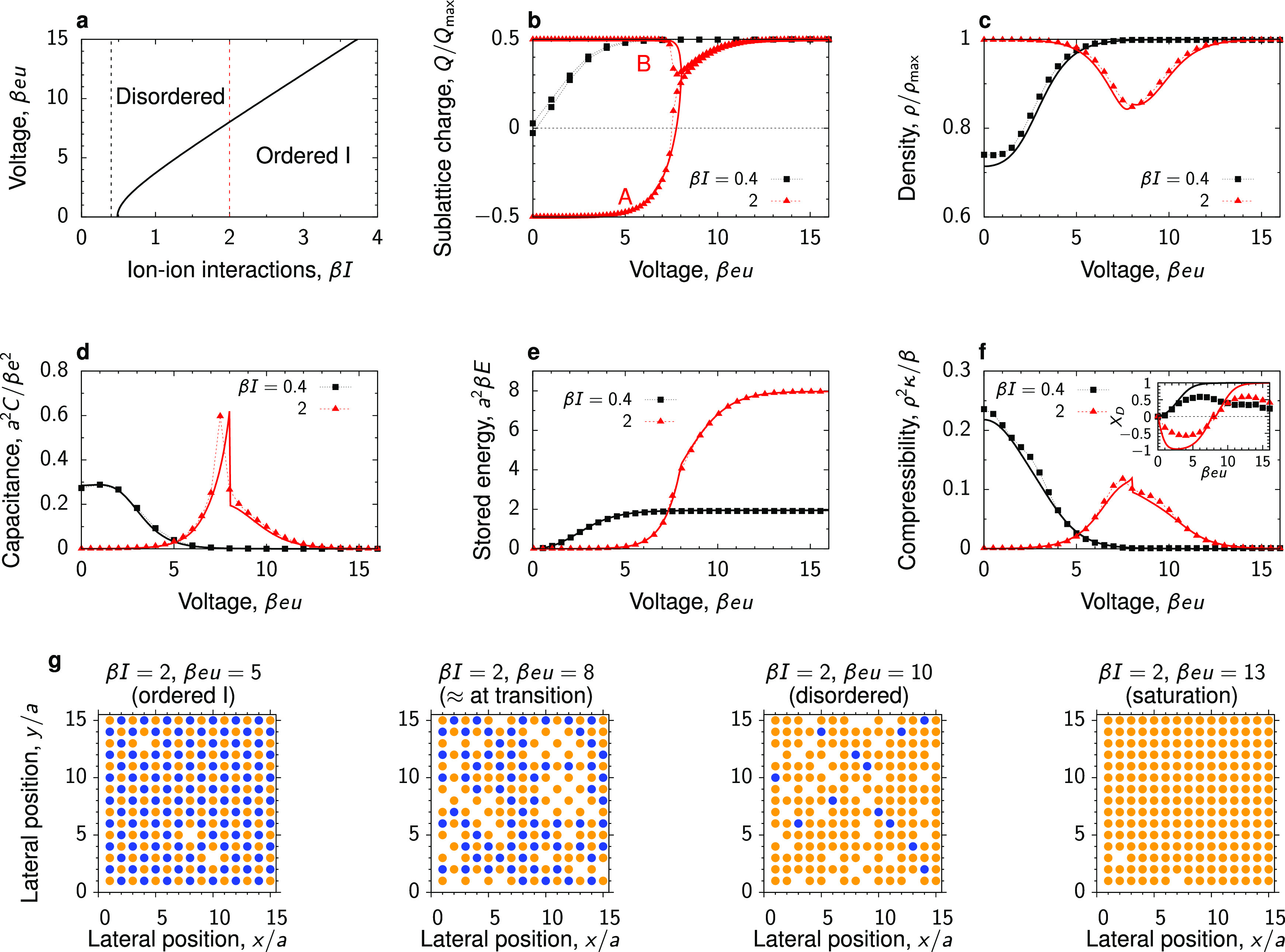
Phase behavior and charging of pores with transfer energy *w* = 0. (a) Phase diagram in the plane of applied voltage *u* and ion–ion interaction energy *I*. The solid line denotes a line of second-order phase transitions separating the disordered phase and the ordered phase (type I). The thin vertical lines show the values of β*I* used in the remaining panels. The diagram has been determined using the Bethe-lattice approach. (b) Charge on sublattices A and B as a function of voltage. The charge on both sublattices is the same for β*I* = 0.4. For β*I* = 2, there is an ordered phase for β*ue* ≲ 8.5, in which the charges on two sublattices have opposite sign but the same magnitude. (c) Total ion density, (d) capacitance, (e) stored energy, and (f) compressibility as functions of applied voltage. The inset in (f) shows the charging parameter *X*_D_ (Equation 8a and 8b). The lines are the Bethe-lattice results, and the symbols denote the results of MC simulations. (g) Snapshots from Monte Carlo simulations with β*I* = 2. For finite-size effects, see Figure S6, and for the results on the honeycomb lattice (*q* = 3), see Figure S7.

Positive transfer energies correspond to ionophilic pores that are occupied by ions at zero potential difference *u*. The charging behavior depends on whether the ions are in the disordered or ordered phase at *u* = 0. In the case of the disordered phase (β*I* = 0.4 in Figure 3), the charging proceeds continuously and the ions remain in the disordered phase at any applied voltage (vertical black dash line in Figure 3a). The ion density increases with *u* due to counterion adsorption, and the capacitance exhibits a small peak close to *u* = 0 before decaying to zero (black lines in Figure 3c,d). Consequently, the stored energy increases and saturates at high voltages (Figure 3e). The charging mechanism consists of a combination of swapping and electrosorption (*X*_D_ > 0, the black line in the inset of Figure 3f). Interestingly, the compressibility has a peak at *u* = 0 but decreases quickly to zero, similarly to the capacitance (Figure 3f). At all potential differences, we found an excellent agreement between the Bethe-lattice approach and the MC simulations (black lines and squares in Figure 3).

If the system at *u* = 0 is in the ordered phase, it undergoes a second-order phase transition to the disordered phase upon increasing the applied voltage (β*I* = 2 in Figure 3). In the ordered phase type I, two sublattices (A and B) are occupied by an equal amount of cations and anions (Figure 3b,g). The ions remain in the ordered state at low voltages so that the accumulated charge and hence the capacitance and stored energy practically vanish in this regime (Figure 3d,e). Upon further increase of voltage, the charging commences by expelling the co-ions from the pore, which implies that the total ion density decreases and the charging parameter *X*_D_ < 0 (Figure 3c and the inset in Figure 3f). Both the capacitance and the compressibility show a peak at the transition (Figure 3d,f). In the MC simulations, the locations of the transitions are slightly shifted toward the ordered phase, compared to the Bethe-lattice predictions. This is likely because the Bethe-lattice approach underestimates the effect of fluctuations and hence overestimates the stability region of the ordered phase.

Interestingly, the stored energy at high applied potentials (viz., in saturation) is about 4 times higher for β*I* = 2 than for β*I* = 0.4 (Figure 3e). This is because for β*I* = 2, the system is in the ordered state at *u* = 0, which persists until a sufficiently high voltage is applied. Thus, the charging is effectively shifted to higher voltages, which leads to higher stored energies. This is similar to charging ionophobic pores, which can provide much higher stored energies compared with ionophilic pores as long as the pore ionophobicity shifts the charging process to higher voltages.^63^

#### Negative Transfer Energy of Ions

A negative transfer energy means that an ion prefers to stay outside of a pore. However, this does not necessarily imply that the pore is empty at zero voltage, as the pore occupancy depends also on the in-pore ion–ion interaction energy β*I*. A high β*I* can compensate the unfavorable transfer energy, thus promoting the ion pairs to go inside the pore. Increasing β*I* therefore increases the pore occupancy.

**Figure 4:**
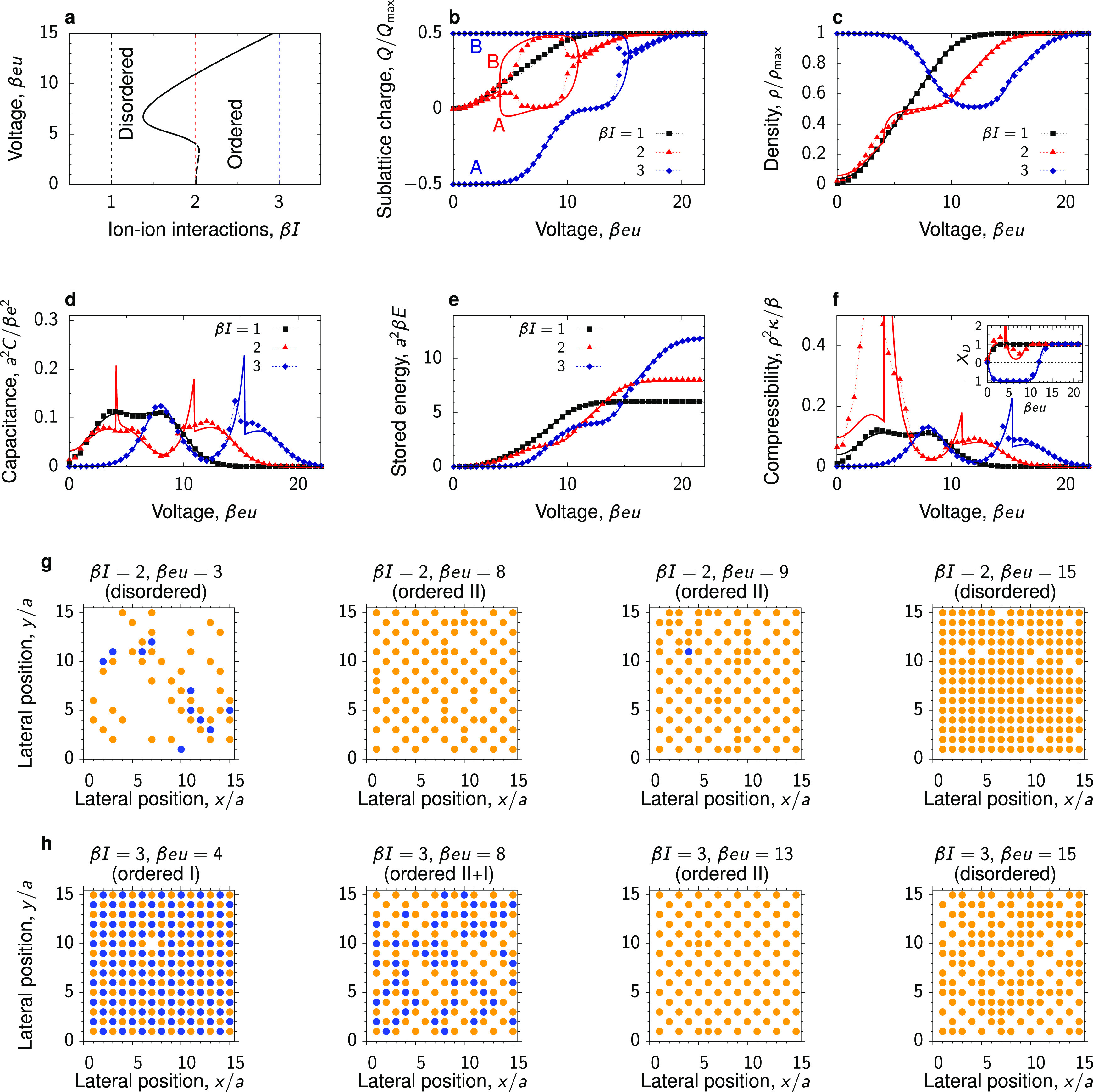
Phase behavior and charging of pores with transfer energy β*w* = −4. (a) Phase diagram in the plane of applied voltage *u* and ion–ion interaction energy *I*. The solid and dash lines denote the lines of second-order and first-order phase transitions, respectively, separating the ordered and disordered phases. The thin vertical lines show the values of β*I* used in the remaining panels. The diagram has been obtained using the Bethe-lattice calculations. (b) Charge on sublattices A and B, (c) total in-pore ion density, (d) capacitance, (e) stored energy, and (f) compressibility as functions of voltage. The inset in (f) shows the charging parameter *X*_D_ (Equation 8a and 8b). The lines are the Bethe-lattice results, and the symbols denote the results of MC simulations. (g, h) Snapshot from Monte Carlo simulations for β*I* = 2 and 3. For finite-size effects, see Figure S8, and for the results on the honeycomb lattice (*q* = 3), see Figure S9.

The case of low β*I* has been studied in ref (48) with the Bethe-lattice approach (for coordination number *q* = 3, see Figure S9). Here, we found a qualitatively similar behavior in the case of a square lattice (*q* = 4; see β*I* = 1, 2 in Figure 4). For β*I* = 1, the charging proceeds continuously with *u*. For β*I* = 2, there are two phase transitions that the system undergoes upon increasing voltage: first from the disordered phase to the ordered one, and then back to the disordered phase. The ordered phase is type II and consists of counterions occupying one sublattice, whereas the other sublattice is free of ions (β*I* = 2 in Figure 4b,g). Interestingly, a similar sequence of the voltage-induced transitions has been reported for an adsorbed layer of butylmethylimidazolium-hexafluorophosphate (BMIM-PF_6_) ionic liquid, forming fluid-like (disordered) and lattice-like (ordered) phases at a graphite electrode.^64^

We found that the MC and Bethe-lattice approaches are again in good agreement, except for the close vicinity of the transitions. For β*I* = 2, the simulations show that the stability of the ordered phase is reduced, compared with the Bethe-lattice results, which is likely due to fluctuations that are not fully accounted for in the Bethe-lattice approach.

The case of high β*I*’s has been omitted in ref (48), but it is interesting in that the type of ordering changes in the course of charging. At zero voltage, the pore is filled with ions, which form type I ordered phase (β*I* = 3 in Figure 4). With increasing the voltage, the counterions remain on their sublattice, while the co-ions gradually leave the pore. Perhaps surprisingly, this process proceeds without a transition. Remarkably, a similar behavior has been observed in molecular dynamics simulations of charging ultranarrow slit nanopores, where the charging proceeded via “melting” of an interface between the two types of “quasi-ordered” ionic liquid states.^65^

Similarly to the case β*w* = 0 (Figure 3), the stored energy in saturation is much higher if the system is in the ordered state at zero voltage (β*I* = 3 in Figure 4e).

### Square, Honeycomb, or Triangular?

We have discussed the charging behavior of superionic liquids using the lattice model, eq 1, with the ions arranged on the honeycomb and square lattices. Although both lattices give rise to a virtually identical qualitative behavior, there are quantitative differences (compare, e.g., Figures 3 and S7). A natural question thus arises as to which of these two types of positional ordering (number of neighbors) could be realized in experimental systems or off-lattice simulations. To address this question, we used the Bethe-lattice approach to compare the free energies (eq 4) of superionic liquids during charging on these two lattices. In addition, we studied charging on a triangular lattice (the number of nearest neighbors *q* = 6), which provides the densest packing of ions. However, the triangular lattice is tripartite, that is, it contains three sublattices and does not allow ordering of ions on two sublattices. Since ordering on the triangular lattice can only be realized at specific concentrations of ions and solvent, here, to avoid these complications, we restrict our considerations to the disordered phase where the sublattice division plays no role. We note that also for the triangular lattice we found a good agreement between the Bethe-lattice calculations and MC simulations (Figures S10 and S11).

**Figure 5:**
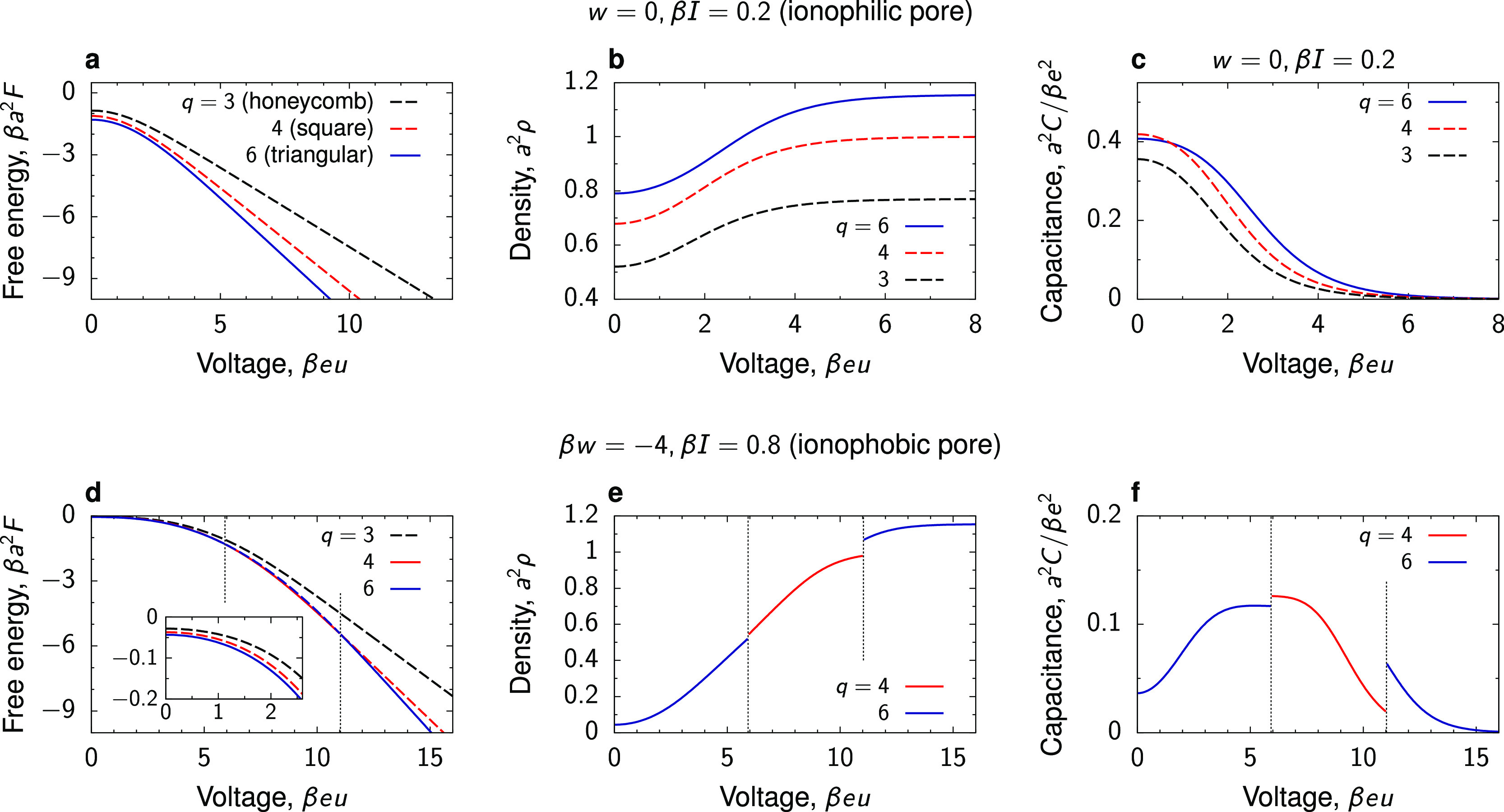
Square, honeycomb, or triangular? (a) Free energy per surface area, *F*; (b) 2D ion density, ρ; and (c) capacitance per surface area, *C*, for honeycomb, square, and triangular lattices obtained by the Bethe-lattice calculations with the coordination numbers *q* = 3, 4, and 6, respectively. The transfer energy of ions *w* = 0 and the ion–ion interaction constant β*I* = 0.2 correspond to an ionophilic pore, occupied by ions at zero voltage. (d–f) Same as (a–c), but for the parameters β*w* = −4 and β*I* = 0.8 corresponding to an ionophobic pore that is nearly free of ions at no applied voltage. The dashed lines denote “metastable” branches having higher free-energy densities. For clarity, only the branches with the lowest free energy are shown in (e, f). The thin vertical lines denote the transformations between the square and triangular ordering.

For an ionophilic pore, the system on the triangular lattice has the lowest free energy at all voltages (Figure 5a). Qualitatively, the ion density and the capacitance behave similarly on all lattices (Figure 5b,c), but, as mentioned, the triangular lattice provides the highest density.

For an ionophobic pore, there is a re-entrant “transition” between the triangular and square lattices (Figure 5d). The square lattice has the lowest free energy for intermediate voltages because, for an increasing ion density, it allows the counterions to avoid the unfavorable direct contact with each other by having fewer nearest neighbors. At sufficiently high voltages, the triangular lattice provides the densest packing and hence the lowest free-energy density. It is interesting to note that hexagonal-symmetry ordering (i.e., the triangular lattice) has been reported in simulations of BMIM-PF_6_ at a graphite electrode.^66^

It is instructive to compare our results with recent 2D off-lattice simulations by Schmickler and Henderson, who revealed spectacular weblike patterns of an ionic liquid in the regime of weak ionophilicities.^67^ These authors also reported on enhanced fluctuations, characteristic of criticality, in some range of parameters. Interestingly, they saw a tendency for ions to form a square lattice at zero voltage, while our Bethe-lattice analysis suggests that hexagonal-symmetry ordering is preferential. We note, however, that our analysis was applied in the regime of the disordered phases (i.e., the same average ion densities on both sublattices), which corresponds to very low ion–ion interaction energies (β*I* = 0.2). The slit width considered in ref (67) was 5*a*, where *a* is the ion diameter. For such wide slits, the coupling constant reaches the value of about 200 *k*_B_*T*, according to eq 2. Moreover, the interactions between the next-nearest and further neighbors become non-negligible (e.g., ϕ(2*a*) ≈ 70 *k*_B_*T*) and hence the results of our simple model cannot be directly compared to the case of ref (67).

## Conclusions

We have studied the charging behavior of superionic liquids in a narrow slit confinement using the recently introduced three-component lattice model.^47,48^ We solved this model exactly on a Bethe-lattice and carried out the corresponding Monte Carlo simulations on the square, honeycomb and triangular lattices. Surprisingly, we found a remarkably good agreement between the analytical results and MC simulations, except for the regions in a close vicinity of second-order transitions. This result is exciting and encourages the application of the analytically tractable Bethe-lattice approach to other systems of electrochemistry and statistical physics.

With MC simulations and Bethe-lattice calculations, we confirmed the rich phase behavior of superionic liquids under an applied voltage, as found in an earlier work.^48^ In addition, we revealed that the energy stored in a pore is a sensitive function of the thermodynamic state. If the in-pore ions are in the ordered state at zero voltage, the system remains in this state until a sufficiently high voltage is applied to break the order, which effectively shifts the charging process to higher voltages and can lead to even a few-fold enhancement of the energy storage. This is similar to the effect of charging ionophobic pores that are free of ions in the nonpolarized state. Indeed, in both cases, the charging is shifted to higher voltages: for ionophobic pores, this is because of an energy barrier for the ions to enter the pore;^63^ for ordered ionic structures inside nonpolarized pores, it is due to the difficulty to break this structure before enriching the pore interior with counterions. Creating a difficulty to do more work to store more energy is a general concept.

Thus, for charging at high voltages, the initial in-pore ordering of ionic liquids might be beneficial for energy storage. Although counterintuitive, this conclusion may appear to be general and model-independent.

## Supporting Information Available

Ion–ion coupling constants; finite-size effects of MC simulations; phase diagram; and charging curves for honeycomb and triangular lattices (PDF)

## References

[ref1] Miller J. R.; Simon P. Materials Science Electrochemical Capacitors for Energy Management. Science 2008, 321, 651–652. 10.1126/science.1158736. 18669852

[ref2] Simon P.; Gogotsi Y. Materials for Electrochemical Capacitors. Nat. Mater. 2008, 7, 845–854. 10.1038/nmat2297. 18956000

[ref3] Béguin F.; Presser V.; Balducci A.; Frackowiak E. Carbons and Electrolytes for Advanced Supercapacitors. Adv. Mater. 2014, 26, 2219–2251. 10.1002/adma.201304137. 24497347

[ref4] González A.; Goikolea E.; Barrena J. A.; Mysyk R. Review on Supercapacitors: Technologies and Materials. Renewable Sustainable Energy Rev. 2016, 58, 1189–1206. 10.1016/j.rser.2015.12.249.

[ref5] Porada S.; Zhao R.; van der Wal A.; Presser V.; Biesheuvel P. Review on the Science and Technology of Water Desalination by Capacitive Deionization. Prog. Mater. Sci. 2013, 58, 1388–1442. 10.1016/j.pmatsci.2013.03.005.

[ref6] Suss M. E.; Presser V. Water Desalination with Energy Storage Electrode Materials. Joule 2018, 2, 10–15. 10.1016/j.joule.2017.12.010.

[ref7] Zhang Y.; Srimuk P.; Aslan M.; Gallei M.; Presser V. Polymer Ion-Exchange Membranes for Capacitive Deionization of Aqueous Media with Low and High Salt Concentration. Desalination 2020, 479, 114331 10.1016/j.desal.2020.114331.

[ref8] Brogioli D. Extracting Renewable Energy from a Salinity Difference Using a Capacitor. Phys. Rev. Lett. 2009, 103, 058501 10.1103/PhysRevLett.103.058501. 19792539

[ref9] Härtel A.; Janssen M.; Weingarth D.; Presser V.; van Roij R. Heat-to-Current Conversion of Low-Grade Heat from a Thermocapacitive Cycle by Supercapacitors. Energy Environ. Sci. 2015, 8, 2396–2401. 10.1039/C5EE01192B.

[ref10] Janssen M.; van Roij R. Reversible Heating in Electric Double Layer Capacitors. Phys. Rev. Lett. 2017, 118, 096001 10.1103/PhysRevLett.118.096001. 28306284

[ref11] Cruz C.; Ciach A.; Lomba E.; Kondrat S. Electrical Double Layers Close to Ionic Liquid-Solvent Demixing. J. Phys. Chem. C 2018, 123, 1596–1601. 10.1021/acs.jpcc.8b09772.

[ref12] Conway B. E. Electrochemical Capacitors: Scientific Fundamentals and Technological Applications; Kluwer, 1999.

[ref13] Chmiola J.; Yushin G.; Gogotsi Y.; Portet C.; Simon P.; Taberna P. L. Anomalous Increase in Carbon Capacitance at Pore Sizes Less Than 1 Nanometer. Science 2006, 313, 1760 10.1126/science.1132195. 16917025

[ref14] Raymundo-Piñero E.; Kierczek K.; Machnikowski J.; Béguin F. Relationship Between the Nanoporous Texture of Activated Carbons and their Capacitance Properties in Different Electrolytes. Carbon 2006, 44, 2498–2507. 10.1016/j.carbon.2006.05.022.

[ref15] Largeot C.; Portet C.; Chmiola J.; Taberna P.-L.; Gogotsi Y.; Simon P. Relation between the Ion Size and Pore Size for an Electric Double-Layer Capacitor. J. Am. Chem. Soc. 2008, 130, 2730–2731. 10.1021/ja7106178. 18257568

[ref16] Kondrat S.; Kornyshev A. Superionic State in Double-Layer Capacitors with Nanoporous Electrodes. J. Phys.: Condens. Matter 2011, 23, 022201 10.1088/0953-8984/23/2/022201. 21406834

[ref17] Rochester C. C.; Lee A. A.; Pruessner G.; Kornyshev A. A. Interionic Interactions in Electronically Conducting Confinement. ChemPhysChem 2013, 16, 4121 10.1002/cphc.201300834. 24311321

[ref18] Goduljan A.; Juarez F.; Mohammadzadeh L.; Quaino P.; Santos E.; Schmickler W. Screening of Ions in Carbon and Gold Nanotubes—A theoretical study. Electrochem. Commun. 2014, 45, 48–51. 10.1016/j.elecom.2014.05.014.

[ref19] Mohammadzadeh L.; Goduljan A.; Juarez F.; Quaino P.; Santos E.; Schmickler W. Nanotubes for Charge Storage—towards an Atomistic Model. Electrochim. Acta 2015, 162, 11–16. 10.1016/j.electacta.2014.12.031.

[ref20] Futamura R.; Iiyama T.; Takasaki Y.; Gogotsi Y.; Biggs M. J.; Salanne M.; Ségalini J.; Simon P.; Kaneko K. Partial Breaking of the Coulombic Ordering of Ionic Liquids Confined in Carbon Nanopores. Nat. Mater. 2017, 16, 1225–1232. 10.1038/nmat4974. 28920938 PMC5702543

[ref21] Kornyshev A. A. The simplest Model of Charge Storage in Single File Metallic Nanopores. Faraday Discuss. 2013, 164, 117–133. 10.1039/c3fd00026e. 24466661

[ref22] Baxter R. J. Exactly Solved Models in Statistical Mechanics; Academic Press, 1982.

[ref23] Lee A. A.; Kondrat S.; Kornyshev A. A. Charge Storage in Conducting Cylindrical Nanopores. Phys. Rev. Lett. 2014, 113, 048701 10.1103/PhysRevLett.113.048701. 25105658

[ref24] Rochester C. C.; Kondrat S.; Pruessner G.; Kornyshev A. A. Charging Ultra-Nanoporous Electrodes with Size-Asymmetric Ions Assisted by Apolar Solvent. J. Phys. Chem. C 2016, 120, 16042 10.1021/acs.jpcc.5b12730.

[ref25] Démery V.; Dean D. S.; Hammant T. C.; Horgan R. R.; Podgornik R. The One-Dimensional Coulomb Lattice Fluid Capacitor. J. Chem. Phys. 2012, 137, 064901 10.1063/1.4740233. 22897305

[ref26] Démery V.; Dean D. S.; Hammant T. C.; Horgan R. R.; Podgornik R. Overscreening in a 1D Lattice Coulomb Gas Model of Ionic Liquids. Europhys. Lett. 2012, 97, 28004 10.1209/0295-5075/97/28004.

[ref27] Démery V.; Monsarrat R.; Dean D. S.; Podgornik R. Phase Diagram of a Bulk 1d Lattice Coulomb Gas. Europhys. Lett. 2016, 113, 18008 10.1209/0295-5075/113/18008.

[ref28] Schmickler W. A Simple Model for Charge Storage in a Nanotube. Electrochim. Acta 2015, 173, 91–95. 10.1016/j.electacta.2015.04.177.

[ref29] Lee A. A.; Kondrat S.; Oshanin G.; Kornyshev A. A. Charging Dynamics of Supercapacitors with Narrow Cylindrical Nanopores. Nanotechnology 2014, 25, 315401. 25026503 10.1088/0957-4484/25/31/315401

[ref30] Frydel D.; Levin Y. Soft-Particle Lattice Gas in One Dimension: One- and Two-Component Cases. Phys. Rev. E 2018, 98, 062123 10.1103/PhysRevE.98.062123.

[ref31] Frydel D. One-Dimensional Coulomb System in a Sticky Wall Confinement: Exact results. Phys. Rev. E 2019, 100, 042113 10.1103/PhysRevE.100.042113. 31770873

[ref32] Liu C.; Yu Z.; Neff D.; Zhamu A.; Jang B. Z. Graphene-Based Supercapacitor with an Ultrahigh Energy Density. Nano Lett. 2010, 10, 4863–4868. 10.1021/nl102661q. 21058713

[ref33] Zhu Y.; Murali S.; Stoller M. D.; Ganesh K. J.; Cai W.; Ferreira P. J.; Pirkle A.; Wallace R. M.; Cychosz K. A.; Thommes M.; et al. Carbon-Based Supercapacitors Produced by Activation of Graphene. Science 2011, 332, 1537–1541. 10.1126/science.1200770. 21566159

[ref34] Tsai W.-Y.; Lin R.; Murali S.; Zhang L. L.; McDonough J. K.; Ruoff R. S.; Taberna P.-L.; Gogotsi Y.; Simon P. Outstanding Performance of Activated Graphene Based Supercapacitors in Ionic Liquid Electrolyte from 50 to 80 °C. Nano Energy 2013, 2, 403–411. 10.1016/j.nanoen.2012.11.006.

[ref35] Chen J.; Li C.; Shi G. Graphene Materials for Electrochemical Capacitors. J. Phys. Chem. Lett. 2013, 4, 1244–1253. 10.1021/jz400160k. 26282137

[ref36] Méndez-Morales T.; Ganfoud N.; Li Z.; Haefele M.; Rotenberg B.; Salanne M. Performance of Microporous Carbon Electrodes for Supercapacitors: Comparing Graphene with Disordered Materials. Energy Storage Mater. 2019, 17, 88–92. 10.1016/j.ensm.2018.11.022.

[ref37] Mendez-Morales T.; Burbano M.; Haefele M.; Rotenberg B.; Salanne M. Ion-Ion Correlations Across and between Electrified Graphene Layers. J. Chem. Phys. 2018, 148, 193812 10.1063/1.5012761. 30307207

[ref38] Lukatskaya M. R.; Mashtalir O.; Ren C. E.; DallAgnese Y.; Rozier P.; Taberna P. L.; Naguib M.; Simon P.; Barsoum M. W.; Gogotsi Y. Cation Intercalation and High Volumetric Capacitance of Two-Dimensional Titanium Carbide. Science 2013, 341, 1502 10.1126/science.1241488. 24072919

[ref39] Naguib M.; Mochalin V. N.; Barsoum M. W.; Gogotsi Y. 25th Anniversary Article: MXenes: A New Family of Two-Dimensional Materials. Adv. Mater. 2014, 26, 992–1005. 10.1002/adma.201304138. 24357390

[ref40] Zhao M.-Q.; Ren C. E.; Ling Z.; Lukatskaya M. R.; Zhang C.; Aken K. L. V.; Barsoum M. W.; Gogotsi Y. Flexible MXene/Carbon Nanotube Composite Paper with High Volumetric Capacitance. Adv. Mater. 2015, 27, 339–345. 10.1002/adma.201404140. 25405330

[ref41] Cuesta J. A.; Sánchez A. General Non-Existence Theorem for Phase Transitions in One-Dimensional Systems with Short Range Interactions, and Physical Examples of Such Transitions. J. Stat. Phys. 2004, 115, 869–893. 10.1023/B:JOSS.0000022373.63640.4e.

[ref42] Cuesta J. A.; Sánchez A. Erratum to: General Non-Existence Theorem for Phase Transitions in One-Dimensional Systems with Short Range Interactions, and Physical Examples of Such Transitions. J. Stat. Phys. 2009, 137, 593–594. 10.1007/s10955-009-9862-6.

[ref43] Montes-Campos H.; Otero-Mato J. M.; Méndez-Morales T.; Cabeza O.; Gallego L. J.; Ciach A.; Varela L. M. Two-dimensional Pattern Formation in Ionic Liquids Confined between Graphene Walls. Phys. Chem. Chem. Phys. 2017, 19, 24505–24512. 10.1039/C7CP04649A. 28890961

[ref44] Girotto M.; dos Santos A. P.; Levin Y. Simulations of Ionic Liquids Confined by Metal Electrodes Using Periodic Green Functions. J. Chem Phys. 2017, 147, 074109 10.1063/1.4989388. 28830185

[ref45] Girotto M.; Colla T.; dos Santos A. P.; Levin Y. Lattice Model of an Ionic Liquid at an Electrified Interface. J. Phys. Chem. B 2017, 121, 6408–6415. 10.1021/acs.jpcb.7b02258. 28590756

[ref46] Girotto M.; Malossi R. M.; dos Santos A. P.; Levin Y. Lattice Model of Ionic Liquid Confined by Metal Electrodes. J. Chem. Phys. 2018, 148, 193829 10.1063/1.5013337. 30307233

[ref47] Dudka M.; Kondrat S.; Kornyshev A.; Oshanin G. Phase Behaviour and Structure of a Superionic Liquid in Nonpolarized Nanoconfinement. J. Phys.: Condens. Matter 2016, 28, 464007 10.1088/0953-8984/28/46/464007. 27624675

[ref48] Dudka M.; Kondrat S.; Bénichou O.; Kornyshev A. A.; Oshanin G. Superionic Liquids in Conducting Nanoslits: A Variety of Phase Transitions and Ensuing Charging Behavior. J. Chem. Phys. 2019, 151, 184105 10.1063/1.5127851. 31731872

[ref49] Blume M. Theory of the First-Order Magnetic Phase Change in UO2. Phys. Rev. 1966, 141, 517 10.1103/PhysRev.141.517.

[ref50] Capel H. W. On the Possibility of First-Order Phase Transitions in Ising Systems of Triplet Ions with Zero-Field Splitting. Physica 1966, 32, 966 10.1016/0031-8914(66)90027-9.

[ref51] Capel H. W. On the Possibility of First-Order Transitions in Ising Systems of Triplet Ions with Zero-Field Splitting II. Physica 1967, 33, 295 10.1016/0031-8914(67)90167-X.

[ref52] Capel H. W. On the Possibility of First-Order Transitions in Ising Systems of Triplet Ions with Zero-Field Splitting III. Physica 1967, 37, 423 10.1016/0031-8914(67)90198-X.

[ref53] Blume M.; Emery V. J.; Griffiths R. B. Ising Model for the λ Transition and Phase Separation in He^3^-He^4^ Mixtures. Phys. Rev. A 1971, 4, 1071 10.1103/PhysRevA.4.1071.

[ref54] Kondrat S.; Georgi N.; Fedorov M. V.; Kornyshev A. A. A Superionic State in Nano-Porous Double-Layer Capacitors: Insights from Monte Carlo Simulations. Phys. Chem. Chem. Phys. 2011, 13, 11359–11366. 10.1039/c1cp20798a. 21566824

[ref55] Kondrat S.; Kornyshev A.; Stoeckli F.; Centeno T. The Effect of Dielectric Permittivity on the Capacitance of Nanoporous Electrodes. Electrochem. Commun. 2013, 34, 348–350. 10.1016/j.elecom.2013.07.009.

[ref56] Jover J. F.; Lugo R.; Toulhoat H.; Simon P.; de Bruin T. Screening Methodology for the Efficient Pairing of Ionic Liquids and Carbonaceous Electrodes Applied to Electric Energy Storage. J. Phys. Chem. C 2014, 118, 864 10.1021/jp409995q.

[ref57] Lee A. A.; Vella D.; Goriely A.; Kondrat S. Capacitance-Power-Hysteresis Trilemma in Nanoporous Supercapacitors. Phys. Rev. X 2016, 6, 021034 10.1103/PhysRevX.6.021034.

[ref58] Limmer D. T.; Merlet C.; Salanne M.; Chandler D.; Madden P. A.; van Roij R.; Rotenberg B. Charge Fluctuations in Nanoscale Capacitors. Phys. Rev. Lett. 2013, 111, 106102 10.1103/PhysRevLett.111.106102. 25166683

[ref59] Forse A. C.; Merlet C.; Griffin J. M.; Grey C. P. New Perspectives on the Charging Mechanisms of Supercapacitors. J. Am. Chem. Soc 2016, 138, 5731–5744. 10.1021/jacs.6b02115. 27031622 PMC4865825

[ref60] Breitsprecher K.; Abele M.; Kondrat S.; Holm C. The Effect of Finite Pore Length on Ion Structure and Charging. J. Chem. Phys. 2017, 147, 104708 10.1063/1.4986346. 28915735

[ref61] Balabajew M.; Balke N.; Bazant M.; Bennewitz R.; Brilliantov N.; de Wijn A. S.; Dey R.; Drummond C.; Dryfe R.; Girault H.; et al. Electroactuators: From Understanding to Micro-Robotics and Energy Conversion: General Discussion. Faraday Discuss. 2017, 199, 525–545. 10.1039/C7FD90031G. 28675404

[ref62] Kowalczyk P.; Ciach A.; Neimark A. V. Adsorption-Induced Deformation of Microporous Carbons: Pore Size Distribution Effect. Langmuir 2008, 24, 6603–6608. 10.1021/la800406c. 18522449

[ref63] Kondrat S.; Kornyshev A. Pressing a spring: What Does It Take to Maximize the Energy Storage in Nanoporous Supercapacitors?. Nanoscale Horiz. 2016, 1, 45–52. 10.1039/C5NH00004A. 32260601

[ref64] Merlet C.; Limmer D. T.; Salanne M.; van Roij R.; Madden P. A.; Chandler D.; Rotenberg B. The Electric Double Layer Has a Life of Its Own. J. Phys. Chem. C 2014, 118, 18291 10.1021/jp503224w.

[ref65] Breitsprecher K.; Holm C.; Kondrat S. Charge Me Slowly, I Am in a Hurry: Optimizing Charge-Discharge Cycles in Nanoporous Supercapacitors. ACS Nano 2018, 12, 9733–9741. 10.1021/acsnano.8b04785. 30088913

[ref66] Merlet C.; Salanne M.; Rotenberg B.; Madden P. A. Imidazolium Ionic Liquid Interfaces with Vapor and Graphite: Interfacial Tension and Capacitance from Coarse-Grained Molecular Simulations. J. Phys. Chem. C 2011, 115, 16613–16618. 10.1021/jp205461g.

[ref67] Schmickler W.; Henderson D. Charge Storage in Two-Dimensional Systems. J. Electroanal. Chem. 2020, 872, 114101 10.1016/j.jelechem.2020.114101.

