# Supplementary material for: Superionic Liquids in Conducting Nanoslits: Insights from Theory and Simulations
Source: J Phys Chem C Nanomater Interfaces. 2021 Mar 1;125(9):4968–76. doi: 10.1021/acs.jpcc.0c10836 (PMC8029497; doi:10.1021/acs.jpcc.0c10836)
Supplement: Supplementary file 1 — jp0c10836_si_001.pdf [file jp0c10836_si_001.pdf]

Supplementary material to "Superionic Liquids in Conducting Nanoslits: Insights from Theory and Simulations"

Yaroslav Groda,[1] Maxym Dudka,[2,3,4] Alexei A. Kornyshev,[5,6] Gleb Oshanin,[7] and Svyatoslav Kondrat[8,9,10]

[1]*Department of Mechanics and Engineering, Belorussian State Technological University, Sverdlova str., 13a, 220006 Minsk, Belarus*

[2]*Institute for Condensed Matter Physics of the National Academy of Sciences of Ukraine, 1 Svientsitskii st., 79011 Lviv, Ukraine*

[3]$\mathbb{L}^4$ *Collaboration & Doctoral College for the Statistical Physics of Complex Systems, Leipzig-Lorraine-Lviv-Coventry, Europe*

[4]*Institute of Theoretical Physics, Faculty of Physics, University of Warsaw, Pasteura 5, 02-093 Warsaw, Poland*

[5]*Department of Chemistry, Molecular Sciences Research Hub, White City Campus, London W12 0BZ,United Kingdom*

[6]*Thomas Young Centre for Theory and Simulation of Materials, Imperial College London, South Kensington Campus, London SW7 2AZ, United Kingdom*

[7]*Sorbonne Université, CNRS, Laboratoire de Physique Théorique de la Matière Condensée, LPTMC (UMR CNRS 7600), 75252 Paris Cedex 05, France*

[8]*Institute of Physical Chemistry, Polish Academy of Sciences, Kasprzaka 44/52, Warsaw, Poland*

[9]*Max-Planck-Institut für Intelligente Systeme, Heisenbergstraße 3, D-70569 Stuttgart, Germany*

[10]*IV. Institut für Theoretische Physik, Universität Stuttgart, Pfaffenwaldring 57, D-70569 Stuttgart, Germany*

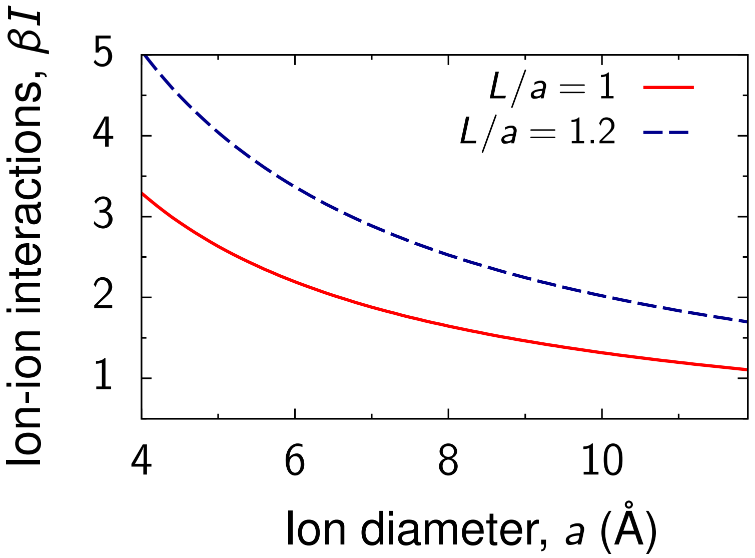

FIG. S1. **Ion-ion interactions.** Relation between the ion diameter a and the coupling constant βI of the Hamiltonian (1) (main text), obtained by using Eq. (2) of the main text. L is the slit width, temperature $T = 300$ K and in-pore dielectric constant $\varepsilon = 5$. The coupling constant I increases with increasing L/a and decreases with increasing the ion diameter a and dielectric constant ε.

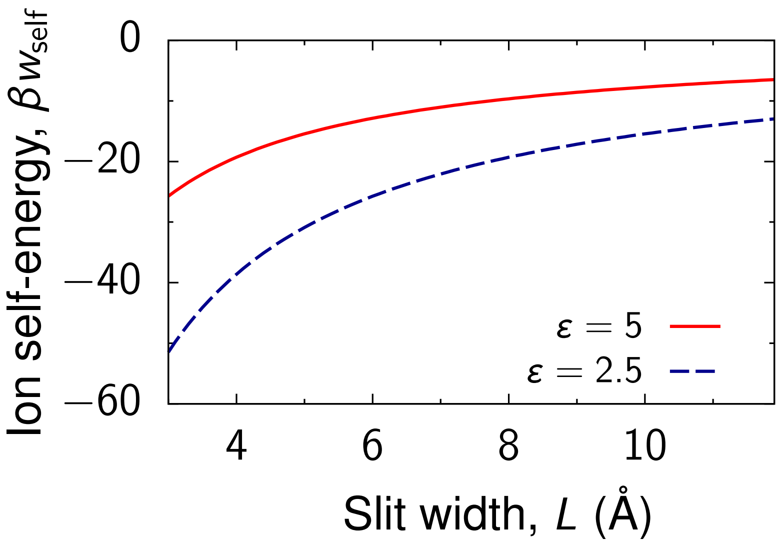

FIG. S2. **Ion self-energy.** Relation between the slit width L and the ion self energy βw_{self} for two values of the in-pore dielectric constant ε. The self energy has been calculated using Eq. (5) of [*J. Phys.: Condens. Matter* **23**, 022201 (2011)]. Temperature $T = 300$ K.

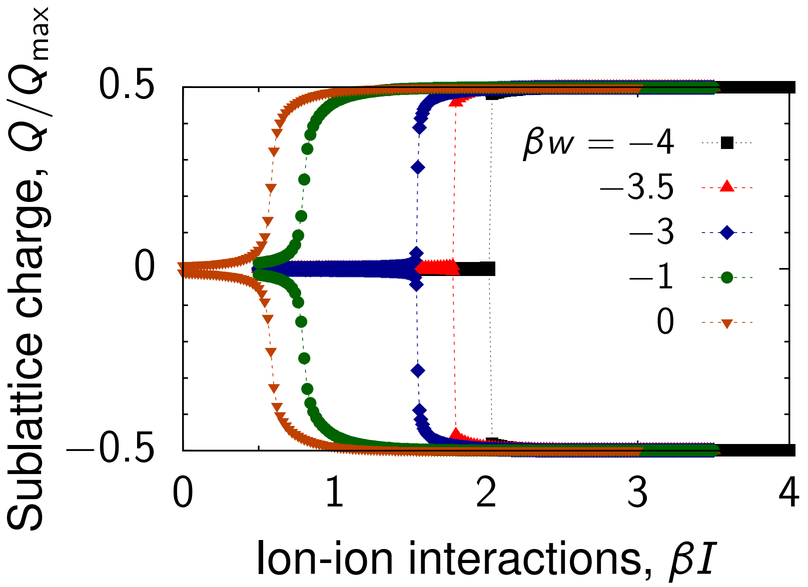

FIG. S3. **Phase transitions in non-polarized electrodes.** Charge on two sublattices for a few resolvation energies. For second-order transitions ($\beta w = 0$ and $\beta I = -1$), the charge on two sublattices changes continuously but becomes steeper as the lattice size increases (Fig. S4a). In the case of first-order transitions ($\beta w = -4$ and $\beta w = -3.5$), there is a jump in the sublattice charge which is practically lattice size independent (Fig. S4b). The value $\beta w = -3$ corresponds approximately to a tricritical point.

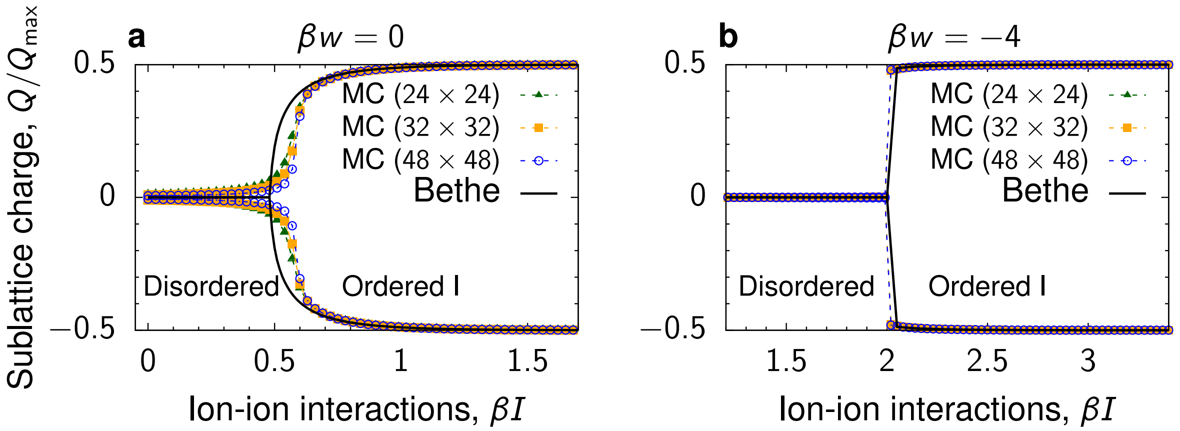

FIG. S4. **Finite-size effects for the phase transitions in non-polarized electrodes.** Charge on two sublattices for the resolvation energy (**a**) $\beta w = 0$ (second-order transition) and (**b**) $\beta w = -4$ (first-order transition) for two lattice sizes. For a second-order transition ($\beta w = 0$), the charge on two sublattices changes continuously but becomes steeper as the lattice size increases. In the case of a first-order transition ($\beta w = -4$), there is a jump in the sublattice charge which is practically lattice size independent. For comparison, the results of the Bethe-lattice calculations with coordination number $q = 4$ are shown by lines.

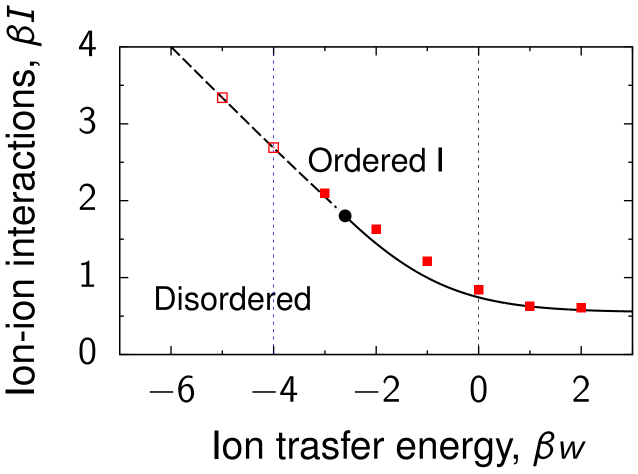

FIG. S5. **Phase diagram of a superionic liquid in a non-polarized ultranarrow slit for the honeycomb lattice.** The results of the Bethe-lattice calculations with coordination number $q = 3$ are shown by lines. The solid lines (filled squares) denote second-order continuous transitions and the dashed lines (open squares) show first-order discontinuous transitions between a disordered phase and the ordered phase I. The thin vertical lines show the values of the transfer energy chosen to study the charging behaviour (Figs. S7 and S9). For the phase diagram on the square lattice, see Fig. 2 in the main text.

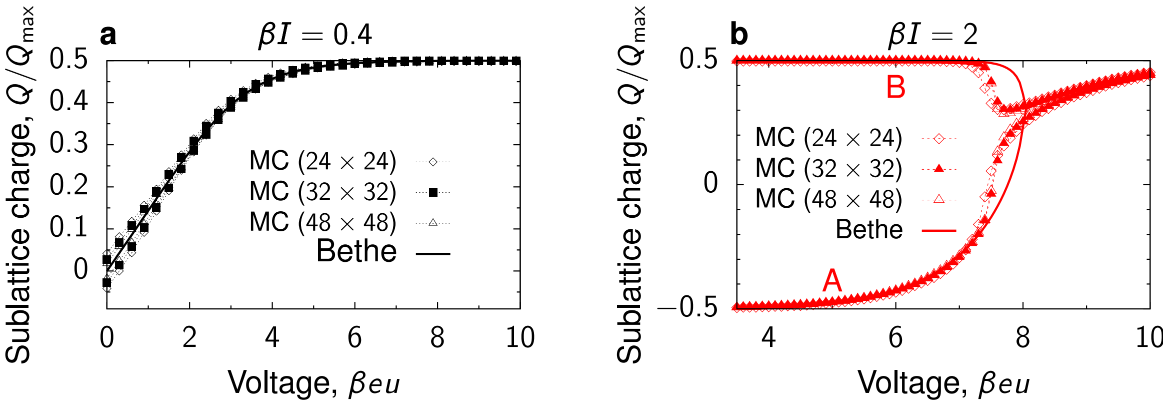

FIG. S6. **Finite size effects for $\beta w = 0$.** Charge on two sublattices for the ion-ion interaction energy (**a**) $\beta I = 0.4$ and (**b**) $\beta I = 2$ for three lattice sizes. The resolvation energy $\beta w = 0$. For comparison, the results of the Bethe-lattice calculations with coordination number $q = 4$ are shown by lines.

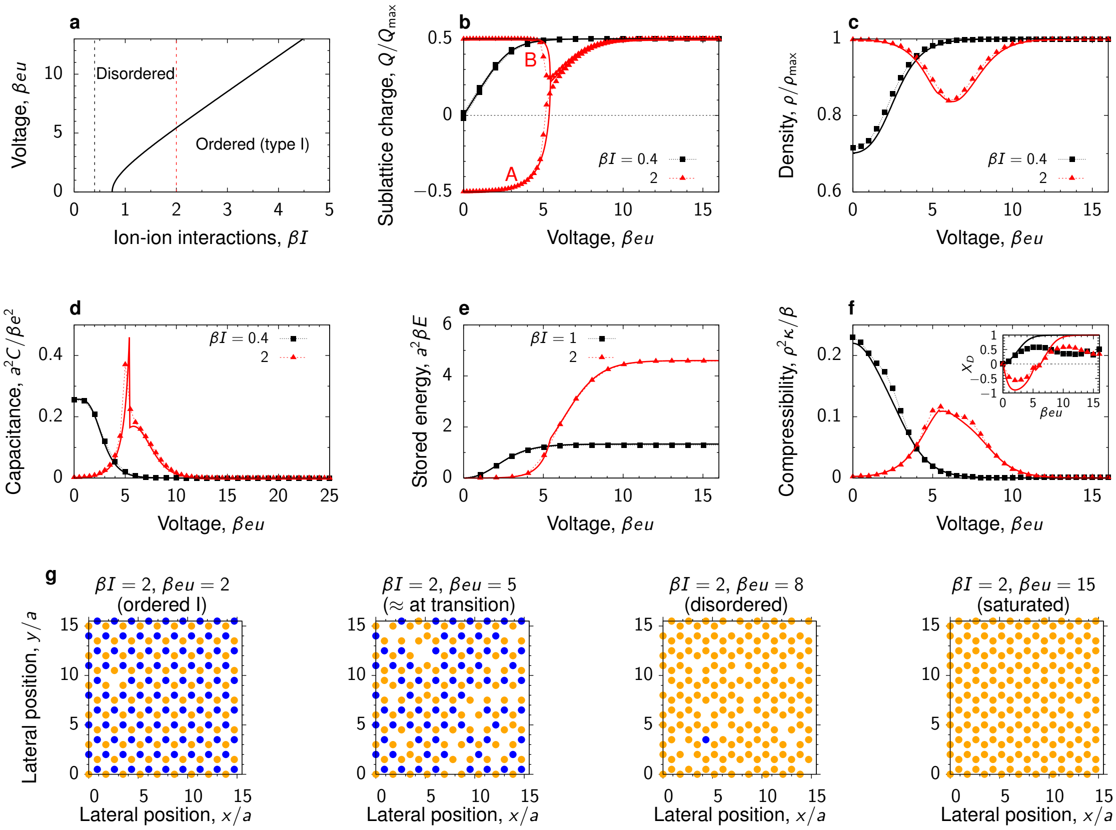

FIG. S7. **Phase behaviour and charging of pores with transfer energy $w = 0$ on the honeycomb lattice.** (**a**) Phase diagram in the plane of applied voltage u and ion-ion interaction energy I. The solid line denotes a line of second-order phase transitions separating the disordered phase and the ordered phase (type I). The thin vertical lines show the values of βI used in the remaining panels. The diagram has been determined using the Bethe-lattice approach. (**b**) Charge on sublattices A and B as a function of voltage. The charge on both sublattices is the same for $\beta I = 0.4$. For $\beta I = 2$, there is an ordered phase for $\beta ue \lesssim 5$, in which the charges on two sublattices have opposite sign but the same magnitude. (**c**) Total ion density, (**d**) capacitance, (**e**) stored energy and (**f**) compressibility as functions of applied voltage. The inset in (**f**) shows the charging parameter X_D, Eq. (8) in the main text. The lines are the Bethe-lattice results and the symbols denote the results of MC simulations. (**g**) Snapshots from Monte Carlo simulations with $\beta I = 2$. The results for the square lattice ($q = 4$) are shown in Fig. 3 of the main text.

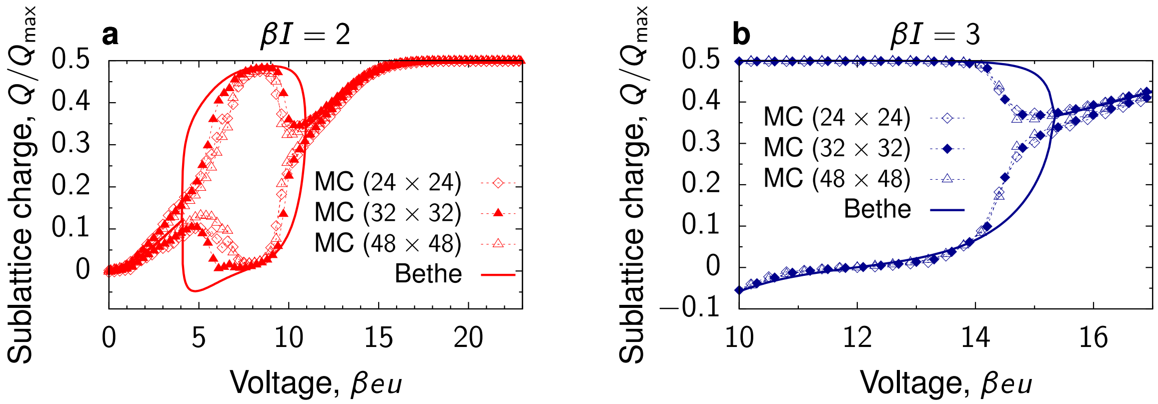

FIG. S8. **Finite-size effects of voltage-induced phase transitions for $\beta w = -4$.** Charge on two sublattices for the ion-ion interaction energy (**a**) $\beta I = 2$ and (**b**) $\beta I = 3$ for three lattice sizes. The resolvation energy $\beta w = -4$. For comparison, the results of the Bethe-lattice calculations with coordination number $q = 4$ are shown by lines.

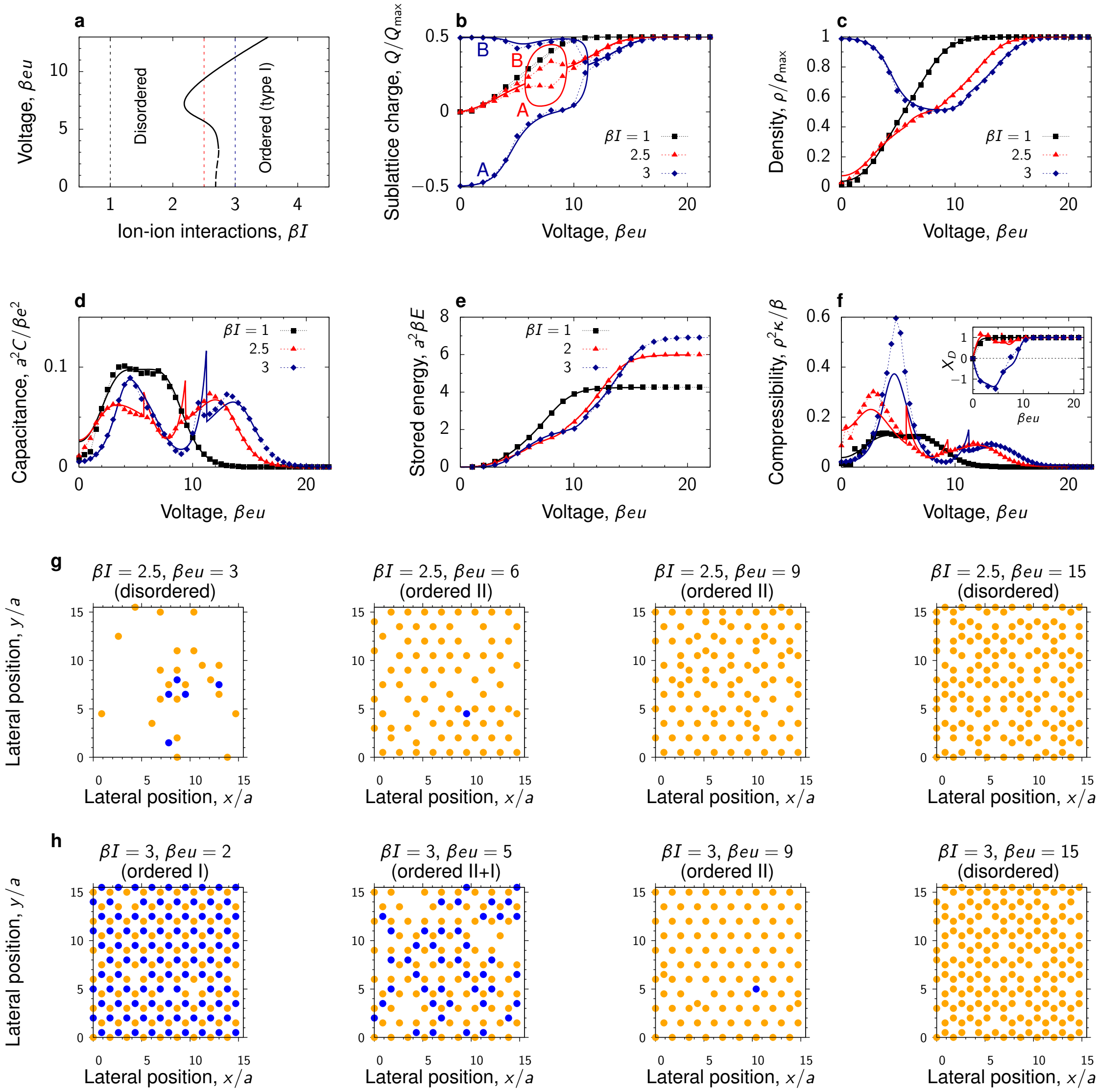

FIG. S9. **Phase behaviour and charging of pores with transfer energy $\beta w = -4$ on the honeycomb lattice.** (**a**) Phase diagram in the plane of applied voltage u and ion-ion interaction energy I. Solid and dash lines denote the lines of second-order and first-order phase transitions, separating the ordered and disordered phases. Thin vertical lines show the values of βI used in the remaining panels. The diagram has been obtained using the Bethe-lattice calculations. (**b**) Charge on sublattices A and B, (**c**) total in-pore ion density, (**d**) capacitance, (**e**) stored energy and (**f**) compressibility as functions of voltage. The inset in (**f**) shows the charging parameter X_D, Eq. (8) in the main text. The lines are the Bethe-lattice results and the symbols denote the results of MC simulations. (**g,h**) Snapshot from Monte Carlo simulations for $\beta I = 2.5$ and $\beta I = 3$. For the results on the square lattice ($q = 4$) see Fig. 4 in the main text.

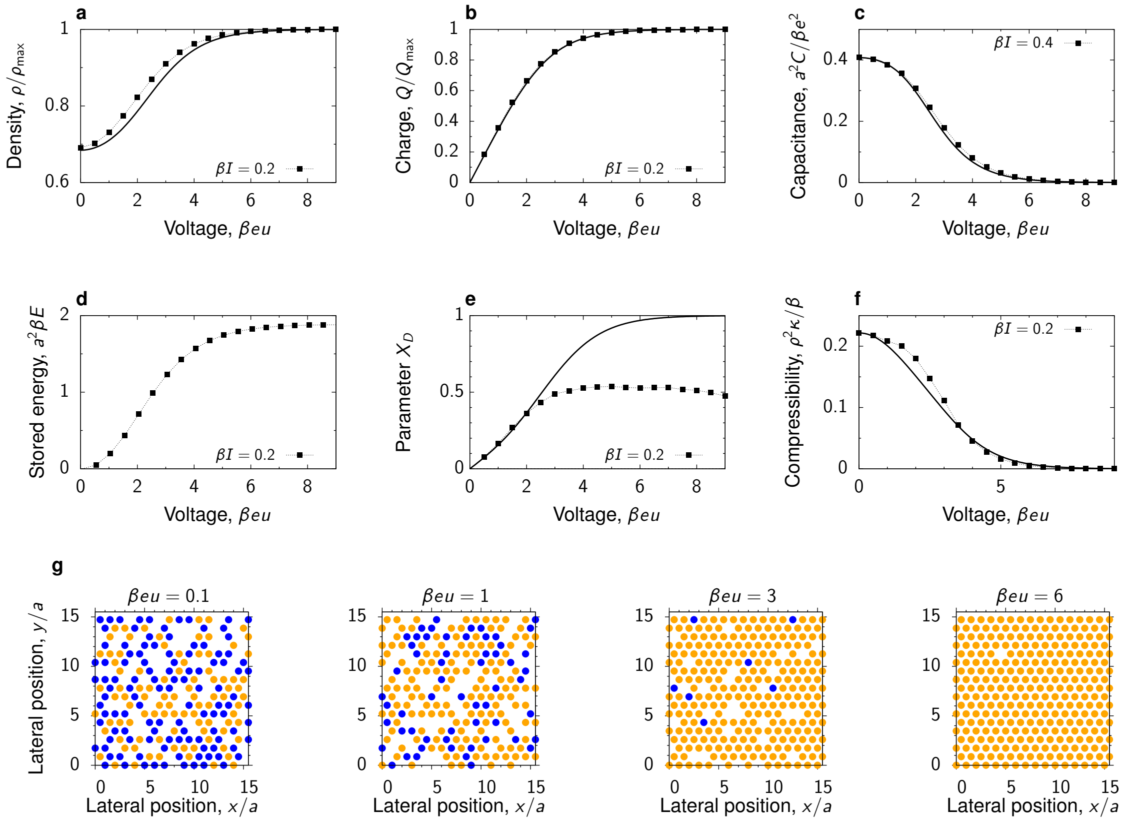

FIG. S10. **Phase behaviour and charging of pores with transfer energy $w = 0$ on the triangular lattice.** (**a**) Ion density, (**b**) charge, (**c**) capacitance, (**d**) stored energy, (**e**) charging parameter X_D and (**f**) compressibility as functions of applied voltage. The lines are the Bethe-lattice results and the symbols denote the results of MC simulations. The origin of the differences in X_D is not cler but they may result from the division of two small numbers (Eq. (8) in the main text), since the capacitance almost vanishes for $\beta eu \gtrsim 6$. (**g**) Snapshots from Monte Carlo simulations. The results for the honeycomb ($q = 3$) and square lattice ($q = 4$) are shown in Fig. S7 and in Fig. 3 of the main text.

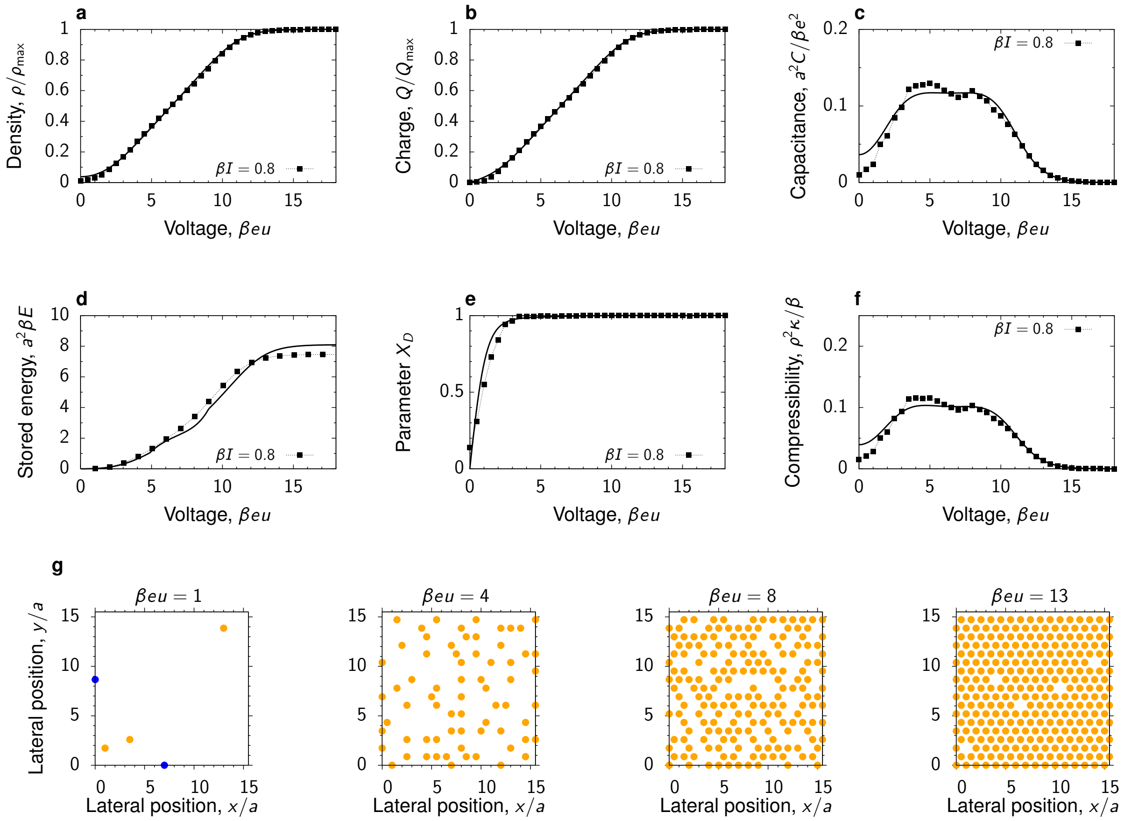

FIG. S11. **Phase behaviour and charging of pores with transfer energy $\beta w = -4$ on the triangular lattice.** (**a**) Ion density, (**b**) charge, (**c**) capacitance, (**d**) stored energy, (**e**) charging parameter X_D and (**f**) compressibility as functions of applied voltage. The lines are the Bethe-lattice results and the symbols denote the results of MC simulations. (**g**) Snapshots from Monte Carlo simulations. The results for the honeycomb ($q = 3$) and square lattice ($q = 4$) are shown in Fig. S9 and in Fig. 4 of the main text.
